# The Function Improved of the Newly Designed Magnetic-End Ureteric Stenting Retrieval Device: A Clinical Prospective Randomized and Control Trial in a Multicenter Study

**DOI:** 10.1155/2022/4107491

**Published:** 2022-04-18

**Authors:** Shaohua Zeng, Bo Liu, Hu Hu, Jian Shi, Ping Qian, Xiaoping Zhang, Kexue Peng, Sixing Yang, Zheng Huang, Tiejun Pan

**Affiliations:** ^1^The First School of Clinical Medicine, Southern Medical University, Guangzhou, China; ^2^Department of Urology, The Sixth Affiliated Hospital of Guangzhou Medical University, Qingyuan People's Hospital, Qingyuan, China; ^3^Department of Urology, General Hospital of Central Theater Command of the People's Liberation Army, Wuhan, China; ^4^Operating Room, Union Hospital, Huazhong Science & Technique University, Wuhan, China; ^5^Department of Urology, Union Hospital, Huazhong Science & Technique University, Wuhan, China; ^6^Department of Urology, Hubei Provincial Rongjun Hospital, Wuhan, China; ^7^Department of Urology, Renmin Hospital of Wuhan University, Wuhan, China; ^8^Wuhan Puffer Medical Instrument Co., LTD, Wuhan, China

## Abstract

**Objective:**

To demonstrate the advantage of our newly designed magnetic ureteric stenting retrieval device over traditional nonmagnetic ureteric stents and other retrieval devices without cystoscopy intervention on clinical application and cost-related outcomes. *Patients and Methods*. A total of 333 patients were recruited into two study groups: magnetic-end ureteral stent (Group A) and conventional ureteral stent (Group B). The effects were evaluated by Ureteral Stent Symptom Questionnaire (USSQ) scores, complications of the indwelling stent, visual analog scale (VAS) pain scores at stent removal, and cost-analysis outcomes between the magnetic ureteric stenting retrieval device and traditional double-J ureteral stent (DJUS) removed by cystoscopy.

**Results:**

The VAS of the pain score of patients undergoing magnetic stent removal with the retrieval device was 2 ± 0.97, whereas that of patients undergoing conventional ureteral stent removal with cystoscopy was 5.76 ± 1.53 (*p* < 0.001). The removal of magnetic stents by a retrieval device proved to be less painful than cystoscopy-mediated stent removal (*p* < 0.001). Obviously, the total cost for the magnetic stent removal was much lower than the conventional ureteral stent removal, although the magnetic stent costs more than the conventional ureteral stent. The improved magnetic stent used in our study showed a remarkable cost saving of 705/111 USD Chinese Yuan (CNY) per patient when compared with the conventional ureteral stent.

**Conclusion:**

We reported the integrated design features of the improved magnetic stent in the world, which was granted a patent in China. USSQ scores and rate of complications in the magnetic stent were as equally acceptable as a conventional stent. Furthermore, successful stent insertion rate reached 100% by both the antegrade and retrograde approaches, and no failure case of magnetic stent removal was reported in our study.

## 1. Introduction 

A ureteral stent was first reported in 1967 and was given the name double-J ureteral stent (DJUS) until 1978, when it was widely used around the world [1]. DJUS was well-accepted for use in surgery for urinary stones and urinary tract obstruction and then was removed by cystoscopy about four weeks later after the operation. Since then, indwelling DJUS and removal, it has become an indispensable integral part of the urological procedure [2]. The side effects of the ureteral stent placement were unavoidable, but limiting indwell time and modifying more new biocompatible material stents, they even added some medications that can effectively reduce patients' urinary symptoms due to the ureteral stent indwelled [3–5]. Nevertheless, the procedure of DJUS removal was still continued using the traditional approach which needs rigid or flexible cystoscopy (mostly rigid cystoscopy in China) to be performed [6, 7]. This additional procedure placed an extra burden on resources, was time-consuming, and caused psychological stress of anxiety and even panic for patients [8]. Cystoscopy itself was often associated with discomfort symptoms during operation [9]. Because indwelling ureteral stent has become a routine and undisputed operation process after operation of kidney and ureteral calculi. So more additional efforts have focused on limiting stent morbidity through decreasing dwell time and modifying stents with various materials impregnated with drugs. Some literature reports removing a ureteral stent using an extraction string without cystoscopy [8, 9]. However, the string exposed outside the genitalia was easy to cause urinary tract infection and sexual activity trouble. Even the complication of displacement with the string was the outstanding shortage for DJUS removal used by the extraction string [10–12].

Recently, a novel DJUS made of polyurethane with a magnet is fixed to its distal part through a string (Magnetic Black-Star®, Urotech GmbH, Achenmuehle, Germany) and extraction of the magnetic-end DJUS by the 15 Fr special retrieval device [13–15]. The method of removing the ureteral stent from the ureter consists of introducing into the bladder a retrieving urinary catheter with a permanent magnet at its tip. Two permanent magnets in the bladder connect, and removal of the catheter follows by extraction. The Magnetic Black-Star® DJUS and retrieval device offers an alternative to conventional ureteral stents in patients. The Black-Star® stent demonstrates an easy quality of life and pain reduction with cystoscopy-free removal in some studies [14–16].

Now, a new designed magnetic-end DJUS was introduced. We called it the new version magnetic-end DJUS which was granted its own patent and approved for use in urology surgery (made in China, Chinese Patent Number: ZL201730073344.X). The key difference was the magnetic structure at the distal of the magnetic-end stent. Nevertheless, we had no data about the application of our improved version magnetic-end DJUS in clinical treatment. Therefore, it was explored through the Ureteral Stent Symptom Questionnaire (USSQ), complications, visual analog scale (VAS) pain scores, and cost-analysis outcomes about our improved version of the magnetic-end DJUS versus a traditional ureteral stent by cystoscopy removal via a prospective randomized trial in a multicenter study.

## 2. Materials and Methods and Patients

### 2.1. Material

#### 2.1.1. Magnetic-End Structure of DJUS and Retrieval Device

**Figure 1:**
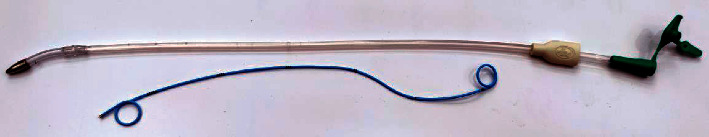
Our improved inversion magnetic-end double-J ureteral stent and the special magnetic retrieval device.

**Figure 2:**
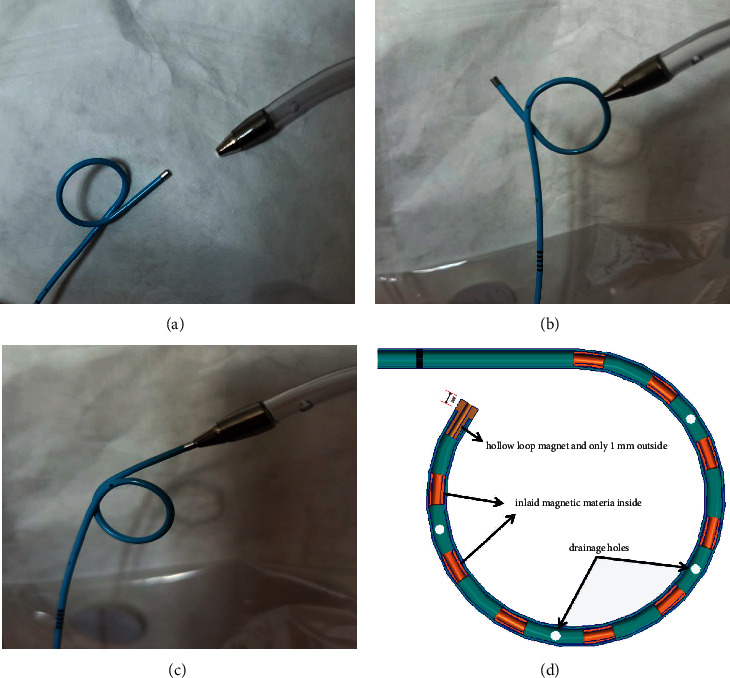
A The clsoe-up images of c and retrieval device. B/C The retrieval device catch the magnetic part (both the hollow loop magnet and the curved part of the stent. D Some thin and soft magnetic materials was inlaid and wrapped inside the curved part, which was almost 5 CM long at the end.

**Figure 3:**
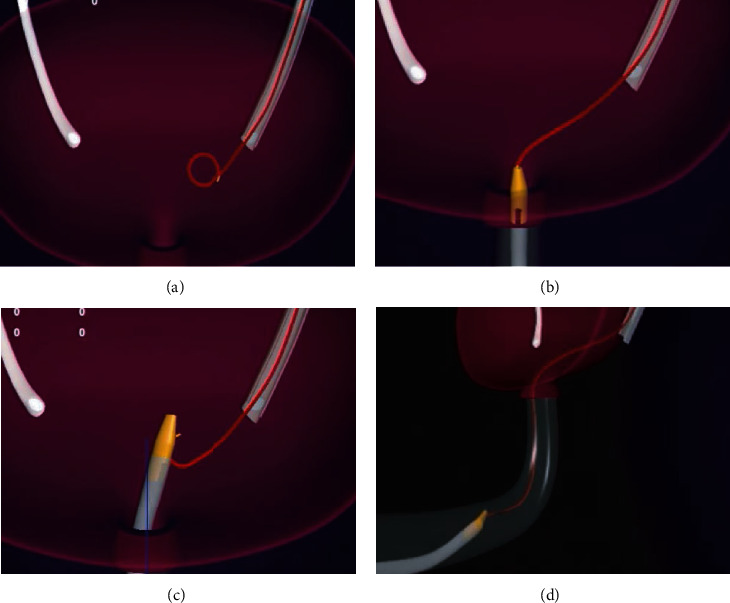
The complete procedure of magnetic-end stent removal are illustrated by four images.

We have introduced a new 6 French diameter magnetic-end DJUS main body made of soft polyurethane which is the same as the previous Magnetic Black Star from Germany. The essential difference of the stent was the magnetic-end structural design at the distal end of the stent. The Magnetic Black-Star magnetic-end structure means a magnetic bead attached to the distal end of the stent by a nylon string. However, our magnetic-end DJUS structure was an integrated design, different from the previous magnetic-end structure (Black Star, Germany). With the same diameter as the stent, a hollow loop magnet, about 1 millimeter long, was closely connected with the distal of the stent to combine the whole structure, integrated as the first part of the improved design (Figure 1). The second component of the improved design was that a thin and soft magnetic material (a magnetic metal belt) was inlaid and wrapped inside the curved part, which was almost 5 CM long at the end (Figure 2). The retrieval device, which resembles a urinary catheter, was inserted into the bladder, and the surgeon conducting the procedure could feel the connection of the two magnets at their respective ends before pulling out the retrieval device and stent together (Figure 3).

### 2.2. Methods and Patients

**Figure 4:**
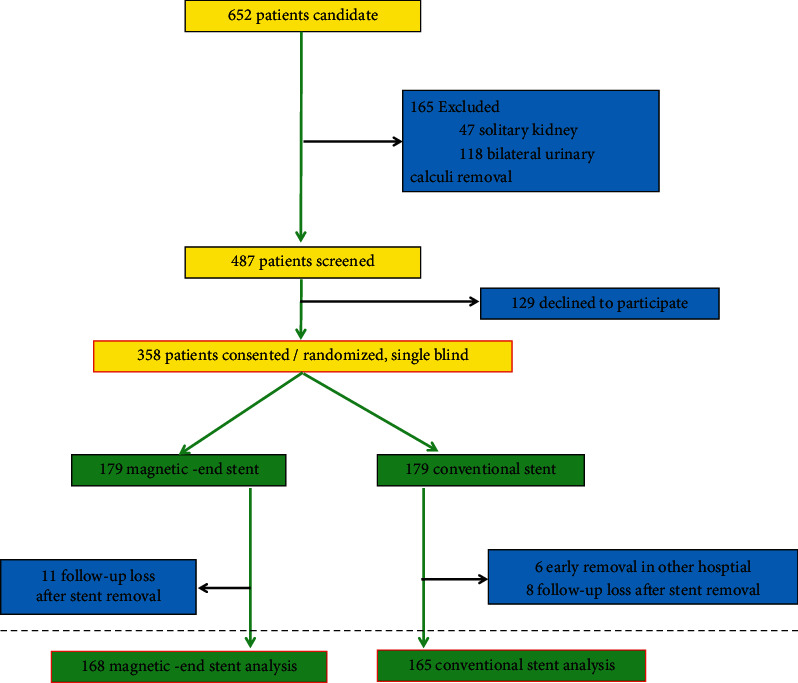
The chart for patients' flow through the study is shown in Figure 4.

There were 652 patients with indwelling DJUS after undergoing routine ureteroscopy or percutaneous nephrolithotripsy for stone disease by experienced surgeons at four urology institutions between May 2018 and April 2021. We excluded 165 cases for solitary kidney and bilateral urinary calculi removal, and 129 patients declined to participate in this study. At last, there were 358 patients enrolled and collected 333 case information (25 cases were follow-up lost) (Table 1). There was a prospective randomized controlled trial at a muticenter. The chart for patients' flow through the study was shown in Figure 4. Following the informed consent procedure, patients were randomly allocated to one of two study groups: magnetic-end ureteral stents (made in China) (Group A) or conventional ureteral stents (Group B). The removal of the magnetic stent was performed in the outpatient clinic, and the conventional stent was removed by a urologist in the cystoscopy room. The review board's approval was obtained.

The USSQ is a validated stent symptom questionnaire that consists of 6 sections and 48 questions [8]. The sections include urinary symptoms, pain, general health, sexual health, and additional problems. We used a validated German version of the USSQ to determine the quality of life of the recipients at postoperative 1 week and 3 weeks in two groups. Adverse events including urinary tract infection, emergency room visit, emergency room visit, phone consultation, readmission, and other complications were monitored in each group. Additionally, we used a pain questionnaire including a visual analog scale (VAS) of pain with scores ranging from 1 to 10 for the extraction of the ureteral stent in two groups. A cost analysis was performed to evaluate the cost per case between the magnetic-end and conventional stent.

### 2.3. Statistical Analysis

Means and standard deviations, or interquartile range (IQR) were used to describe continuous variables. The frequencies and proportions of categorical variables were reported as percentages. The characteristics of patients were analyzed using Student's *t*-test or the Mann-Whitney rank-sum test. Proportions were compared using the chi-square test. A *p* value of <0.05 was considered indicative of statistically significant differences. SPSS 22.0 for windows (IBM SPSS version 22.0, IBM, Armonk, NY, USA) was used for all statistical analyses.

## 3. Results

### 3.1. Patient Demographics and Clinical Information

The clinical information and demographic characteristics were not statistically different between each group in this prospective randomized control clinical trial (Table 1).

### 3.2. The USSQ Scores of the Magnetic Stent Group and the Conventional Stent Group

**Figure 5:**
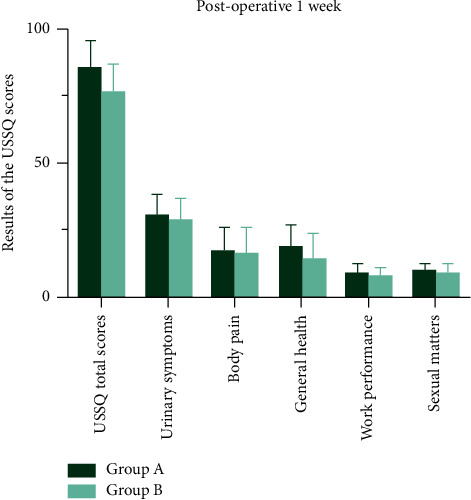
Urinary index scores measured by USSQ in patients with a stent indwelling were not significantly different between groups at postoperative 1 week.

**Figure 6:**
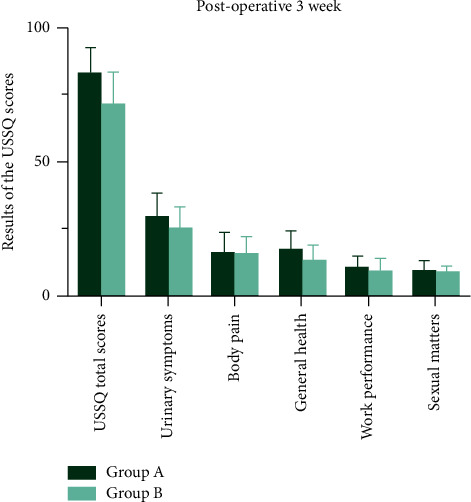
Urinary index scores measured by USSQ in patients with a stent indwelling were not significantly different between groups postoperative at 3 week.

Urinary index scores measured by USSQ in patients with a stent indwelling were not significantly different between groups (Table 2 and Figures 5–6).

### 3.3. The Complications Associated of the Magnetic Stent Group and the Conventional Stent Group

The complications associated with the ureteral stent indwelling includes urinary tract infection, emergency room visits, phone consultations, readmission, stent-related sexual annoy, analgesics usage and other rare complications. However, we observed no difference in complication incidence between the two groups (Table 3).

### 3.4. The VAS Pain Scores of the Magnetic Stent Group and the Conventional Stent Group

There was significantly less pain resulting from stent removal in the magnetic stent group as assessed by the VAS. The magnetic stent removal with the retrieval device led to a VAS pain score of 2 ± 0.97, whereas a VAS pain score of 5.76 ± 1.53 was observed in patients undergoing conventional ureteral stent removal with cystoscopy (*p* < 0.001) (Table 4). Obviously, the overall cost for the magnetic stent removal was lower than the conventional ureteral stent removal, although the magnetic stent costs more than the conventional ureteral stent.

### 3.5. Cost-Analysis of the Magnetic Stent Group and the Conventional Stent Group Stent per Case

The cost of conventional stent removal by cystoscopy was ¥1700/267 USD in Chinese Yuan (¥CNY), including the cost of a stent (¥800/126 USD) and the operation fee for stent removal by cystoscopy (¥900/141 USD). The cost of magnetic stent removal was ¥995/156 USD in Chinese Yuan (CNY), including the cost of the stent (¥920/145 USD) and the operation fee for stent removal by retrieval device in outpatient (¥75/12 USD). The improved magnetic stent used in our study showed a remarkable cost saving of ¥705/111 USD in Chinese Yuan (CNY) per patient (Table 5) when compared with the conventional ureteral stent.

## 4. Discussion

The ureteral stent removal by cystoscopy was a necessary procedure for conventional stent indwelling DJUS after undergoing routine ureteroscopy or percutaneous nephrolithotripsy. However, stent removal by cystoscopy has been associated with discomfort such as pain. For men, especially old men, cystoscopy removal of the stent tube was a terrible experience [17]. An alternative noncystoscopy removal for the ureteral stent was the use of a string attached to the distal part of the DJUS. The end of the string was hanging out of the urethra. Pulling the string instead of cystoscopy manipulation was used for ureteral stent removal [12, 18]. Common complications including urinary infection, urinary leakage, and sexual intercourse distress occurred if the DJUS was needed to indwell longer time [12, 18]. The high rate of stent dislodgement was almost up to 15%, which was a shortcoming of extraction strings [12, 19]. The well-accepted “ideal” ureteral stent was biodegradable without removal and noncytotoxicity for the body, but still much progress has been made in the physical characteristics and biocompatibility of the biomaterials in vitro until now [20]. Recently, many studies have reported other nonendoscopic techniques for stent removal such as magnetic-end ureteral stent which can be used to prevent urological complications for kidney transplantation, children's urinary calculus, and ureteropelvic junction obstruction [13, 14]. A magnetic ureteral stent (Black-Star®, made in Germany) was a typical application for clinical treatment [13, 14, 16]. The removal of magnetic stents using a retrieval catheter proved to be less painful than using cystoscopy [16, 21].

Our results showed that a novel improved magnetic stent indwelling presented no statistical differences in USSQ scores when compared with a conventional stent. However, significantly lower pain scores were observed in the magnetic stent removal group than in the conventional stent removal group. The overall cost of the stent in our study showed a remarkable cost saving of ￥705/111 USD Chinese Yuan (CNY) per patient. Our results basically coincide with the results from studies of Black-Star magnetic-end ureteral stent removal versus conventional stent removal in previous reports [14]. Nevertheless, our novel improved magnetic-end ureteral stent had significant differences from the Black-Star stent in magnetic-end structure. The great advantage was the integrated design which combined the hollow annular magnet (same diameter as a stent) with the distal of the stent instead of a magnetic bead linked by a nylon line in the Black-Star stent.

However, an antegrade stent placement failure rate was 34% (16 cases failed in all 47 cases) in pyeloplasty with an antegrade approach used by Black-Star [14]. Because the rigid magnetic-end metal bead was separated from the distal of stent, it cannot pass through the UVJ smoothly sometimes. Furthermore, the single metal bead would be stuck in the place of ureter straitness or contortion by antegrade approach insertion. In our new improved integrated design of the magnetic-end structure, both antegrade (percutaneous nephrolithotripsy) and retrograde (ureteroscopic lithotripsy) stent insertions were all successful in the 168 cases studied.

Likewise, there were three reasons for the failure of noncystoscopy stent removal in literature reports. Firstly, the magnetic bead was stuck in the bladder diverticulum. In addition, significant and thick encrustation on the surface of the magnetic bead led to impossible bead contact with the magnetic retrieval device. Thirdly, the presence of a large median lobe in severe prostate hypertrophy was considered as a barrier to hinder retrieval device catch magnetic bead [13, 14]. We have effectively solved the problem of failed noncystoscopy stent removal on the Black-Star stent with the help of the new improved integrated magnetic design. With the same diameter as the stent, a hollow annular magnet, about 1 millimeter long, was closely connected with the distal of the stent to combine the whole structure integrated. The smaller the surface area of the metal magnetic ring is, the less likely it is to develop encrustation around the surface. And the design can also perfectly solve the problem of the magnetic bead getting stuck in the bladder diverticulum. Moreover, we have inlaid and wrapped the thin and soft magnetic material (magnetic metal belt) inside the curved part which is almost 5 CM long at the end of the stent. This highlight design expands the length of the magnetic part, including the hollow annular, to make the retrieval device catch the magnetic part (both the hollow annular magnet and the 5 cm magnetic metal belt inside) more easily at the end of the stent in the bladder. With the help of expanding the length of the magnetic part, we achieved 100% successful stent removal in all prostate hypertrophy cases, even in patients with a large median lobe. Although the removal of these cases takes more time than others. In addition, the symptoms of indwelling these stents in patients with prostatic hypertrophy need further attention and follow-up to be more convincing by showing the accompanying symptoms of the patient during treatment.

In addition, the present results were encouraging for the reason that the use of the magnetic stent removal realized cost reduction and pain relief. More importantly, this procedure can bring the patients more positive and relaxed emotions and higher compliance. However, the better psychological care due to the improved magnetic stent removal could not be demonstrated in our data. Another limitation of our study was that the stent removal operation was performed by different surgeons from different centers. The effect of different procedure times on patient outcomes cannot be ignored.

## 5. Conclusion

We report here for the first time a clinical study on a new improved magnetic-end stent device, which was granted a patent in China (Chinese Patent Number: ZL201730073344.X). USSQ scores and complication rates in the magnetic stent were as equally acceptable as a conventional stent. Use of our stent resulted in an estimated savings of 705/111 USD Chinese Yuan (CNY) per patient. Pain perception in noncystoscopy removal was significantly less than in conventional removal. Furthermore, due to the new improved integrated design of the magnetic-end structure, the successful stent insertion rate reaches 100% by both antegrade and retrograde approaches. And no failure case of magnetic stent removal was reported in our study.

## Figures and Tables

**Table 1:** Patient demographics and clinical information.

	Magnetic stent group (*n* = 168)	Conventional stent group (*n* = 165)	*P* value
Gender, *n* (%)			
Male	118 (70.2)	115 (69.7)	0.912
Female	50 (29.8)	50 (30.3)	0.892
Age, years (mean ± SD)	45.7 (±13.5)	49.5 (±14.6)	0.789
BMI, kg/m^2^ (mean ± SD)	22.1 (±2.5)	23.3 (±3.1)	0.754
Stone size, mm (mean ± SD)	12.1 (±1.7)	11.8 (±2.5)	0.658
Stone location, *n* (%)			0.472
Ureter	109 (64.9)	113 (68.5)	
Kidney	43 (25.6)	38 (23.0)	
Ureter and kidney	16 (9.5)	14 (8.5)	
Surgery type, *n* (%)			0.344
Flexible ureteroscopy	97 (57.8)	109 (66.1)	
Rigid ureteroscopy	55 (32.7)	50 (30.3)	
PCNL	16 (9.5)	6 (3.6)	

**Table 2:** Results of the USSQ scores between two groups.

	Postoperative 1 week			Postoperative 3 week		
	Group A	Group B	*P*value	Group A	Group B	*P* value
USSQ total scores (mean ± SD)	85.7 (±9.9)	76.5 (±10.3)	0.351	83.1 (±9.4)	71.6 (±11.8)	0.287
Urinary symptoms (mean ± SD)	30.5 (±7.5)	28.6 (±7.8)	0.556	29.6 (±8.5)	25.4 (±7.3)	0.344
Body pain (mean ± SD)	17.4 (±8.4)	16.8 (±9.1)	0.552	16.1 (±7.1)	15.6 (±6.2)	0.763
General health (mean ± SD)	18.9 (±7.8)	14.4 (±9.1)	0.254	17.4 (±6.5)	13.3 (±5.3)	0.819
Work performance (mean ± SD)	8.9 (±3.1)	7.8 (±.8)	0.845	10.7 (±3.9)	9.1(±4.5)	0.841
Sexual matters (mean ± SD)	9.9 (±2.3)	8.6 (±3.8)	0.709	9.4 (±3.3)	8.2 (±2.5)	0.769

Comparisons of the USSQ scores between two groups at 1 and 3 weeks postoperative follow-up.

**Table 3:** Complications associated with the different stent groups.

	Magnetic stent group	Conventional stent group	*P* value
UTI, *n* (%)	17 (10.1)	15 (9.0)	0.922
ER visit, *n* (%)	25 (14.9)	17 (10.3)	0.815
Phone consultation, *n* (%)	42 (25.1)	38 (23.0)	0.963
Readmission, *n* (%)	7 (4.2)	4 (2.4)	0.811
SR sexual annoyed, *n* (%)	3 (1.8)	4 (2.4)	0.917
Analgesics, *n* (%)			
YES	20 (12.1)	15 (9.0)	0.584
NO	9 (5.4)	9 (5.5)	0.915
Other, *n* (%)			
Acute urinary retention	2 (1.2)	0 (0)	0.804
Diarrhea	3 (1.8)	1 (0.6)	0.775
Allergic reaction	2 (1.2)	0 (0)	0.854
Constipation	7 (4.2)	5 (3.0)	0.785

Comparisons of the complications associated with different stents indwelling between magneted stent group and conventional stent group.

**Table 4:** VAS pain scores at stent removal moment via different methods.

	Magnetic stent group		Conventional stent group		*P* value
	Median	Mean (SD)	Median	Mean (SD)	
Pain during removal (VAS)	2	2.1 (±0.97)	6	5.76 (±1.53)	0.001

**Table 5:** Cost-analysis of two different group stents per case (CNY, ¥).

	Cost of magnetic group	Cost of conventional group
Cost of stent	920	800
Cystoscopy removal	0	900
Outpatient removal	75	0
Total cost, ¥	995	1700

CNY = USD 995 = 156, 1700 = 267.

## Data Availability

The data used to support the findings of this study are available from the corresponding author upon request.
